# Influence of Sex and Body Composition on Aerobic Capacity in Normal Weight Lean, Normal Weight Obese, and Obese Phenotypes

**DOI:** 10.3390/ijerph22010103

**Published:** 2025-01-14

**Authors:** Sam R. Emerson, Samantha Hart, Christina M. Sciarrillo, Travis Eden, Tyler J. Godsey, Harrison Smith, Ashley Keller, Bryant H. Keirns

**Affiliations:** 1 Department of Nutritional Sciences, Oklahoma State University, Stillwater, OK 74078, USAtravis.eden@okstate.edu (T.E.); tylgods@okstate.edu (T.J.G.);; 2 National Institute of Diabetes and Digestive and Kidney Diseases, National Institutes of Health, Bethesda, MD 20892, USA; christina.sciarrillo@nih.gov; 3 Department of Nutrition and Health Science, Ball State University, Muncie, IN 47306, USA; bryant.keirns@bsu.edu

**Keywords:** normal weight obesity, body composition, sex differences, aerobic capacity, VO_2peak_, body mass index, BMI, visceral adipose tissue, lean mass, obesity

## Abstract

Normal weight obesity (NWO) is a body composition phenotype that is associated with increased cardiometabolic risk and is characterized by a normal weight body mass index but elevated body fat. The purpose of this study was to determine sex differences in aerobic capacity across body composition phenotypes, including normal weight lean (NWL), NWO, and traditional obesity (OB). We recruited 60 participants according to three body composition phenotypes: NWL (n = 10 females, n = 10 males), NWO (n = 10 females, n = 10 males), and OB (n = 10 females, n = 10 males). Measurements included fasting metabolic risk factors, body composition X-ray scan, and peak exercise test on a cycle ergometer to determine aerobic capacity (VO_2peak_). Across groups, males (34.5 ± 11.7 mL/kg/min) exhibited greater VO_2peak_ than females (28.8 ± 8.8 mL/kg/min; *p* = 0.04). There were no differences in VO_2peak_ between sexes within the same body composition phenotype, but NWL (42.7 ± 9.0 mL/kg/min) exhibited greater VO_2peak_ than NWO (27.9 ± 4.4 mL/kg/min; *p* < 0.0001) and OB (24.4 ± 7.3 mL/kg/min; *p* < 0.0001). VO_2peak_ was inversely correlated with relative body fat in the full sample (r = −0.67; *p* < 0.0001), but was stronger in males (*r* = −0.78; *p* < 0.0001) than females (*r* = −0.53; *p* = 0.0028). Visceral adipose tissue was not significantly correlated with VO_2peak_ in the full sample (*r* = −0.25; *p* = 0.05) or in males (*r* = −0.23; *p* = 0.25), although they were inversely correlated in females (*r* = −0.36; *p* = 0.048). Our results suggest low aerobic capacity in both men and women with NWO, similar to men and women with OB. The relationship between body composition and aerobic capacity is strong across body composition phenotypes, but appears to be more consistent in females than males. For healthcare professionals aiming to lower cardiometabolic risk, attention should be given to improving aerobic fitness in both men and women with elevated body fat, including those with NWO.

## 1. Introduction

Cardiovascular disease is one of the most prevalent health issues in developed nations, and it has remained the leading cause of death in the United States for over a century [1]. Although the annual mortality rate for cardiovascular disease has declined, in 2022, it contributed to 21% of deaths in the United States [2]. Aerobic capacity is the measure of the maximum or peak volume of oxygen (VO_2peak_) the body utilizes during exercise, and this metric is significant for reflecting cardiorespiratory capacity and health. Evidence has firmly established the link between cardiovascular disease and aerobic capacity. For example, lower aerobic capacity may be a stronger predictor of mortality than other risk factors like hypertension, type 2 diabetes, smoking, and cholesterol levels [3,4]. Furthermore, every 1 mL/kg/min increase in VO_2peak_ is associated with a 15% decrease in risk of death [4,5]. It is evident that aerobic capacity is crucial for cardiovascular health, requiring the recognition of individuals or populations that may have reduced aerobic capacity.

Obesity, defined by elevated adiposity but traditionally classified as a body mass index (BMI) of 30.0 kg/m^2^ or greater, has been widely associated with a plethora of negative health conditions, including increased cardiovascular disease risk [6]. Additionally, mounting evidence has established the relationship between obesity and reduced aerobic capacity [6,7]. Excess adiposity increases the energy cost of physical activity, reducing overall efficiency during exercise [8]. Additionally, obesity impairs cardiovascular and respiratory function by increasing cardiac workload and reducing pulmonary compliance, leading to diminished oxygen delivery and utilization during exercise [9]. Further, obesity-induced systemic inflammation and mitochondrial dysfunction negatively impact muscle oxidative capacity, contributing to reduced aerobic performance [10,11]. However, BMI, as a construct for representing increased adiposity, has flaws. These concerns include the misclassification of risk in some populations, resulting in either over- or under-estimation of risk. Individuals with normal weight obesity (NWO) are an example of BMI under-estimating risk. NWO describes individuals with a normal-range BMI (18.5–24.9 kg/m^2^) with high relative body fat [12]. While these individuals have maintained a normal-weight BMI, NWO is associated with an increased risk of cardiovascular disease mortality, especially if the excess relative fat tissue is distributed as visceral or central fat [13,14].

Current knowledge of NWO’s aerobic capacity is limited. For example, some studies that explore differences between NWO and normal-weight lean (NWL) individuals have assessed aerobic capacity via submaximal-heart-rate-based tests rather than a progressive test to volitional fatigue [15]. Additionally, previous reports have compared NWO to combined overweight and obesity groups, which may not be distinct enough from NWO to draw conclusions regarding aerobic capacity in NWO compared to other body composition profiles [16]. To address these inconsistencies, a recent study was conducted in our laboratory to examine aerobic capacity differences between healthy controls and NWO individuals, as well as individuals classified as metabolically healthy obese, another relevant and understudied body composition phenotype [17]. Our results [17], as well as others [15,18], suggest lower aerobic capacity in people with NWO, aligning with the concept of increased cardiovascular disease risk in this population. However, our data suggest that there is a strong relationship between body composition parameters and aerobic capacity across body composition phenotypes and sexes [17]. Given (1) documented sex differences in body composition [19], (2) sex differences in aerobic capacity [20], and (3) evidence showing the prevalence of NWO and related cardiovascular disease risks vary based on sex [13,21], whether impaired aerobic capacity is mediated by sex in NWO warrants examination.

The purpose of this study was to determine sex differences in aerobic capacity across body composition phenotypes, including NWL, NWO, and traditional obesity (OB). We hypothesized that NWL would exhibit greater aerobic capacity than NWO and OB, primarily due to less body fat in NWL, and that this trend would be observed in both female- and male-specific analyses. We also hypothesized that females would exhibit a lower aerobic capacity than males within each body composition phenotype, including NWO.

## 2. Materials and Methods

### 2.1. Participant Characteristics

Participants (N = 60; ages 18–50 years) were recruited through email and flyers distributed at Oklahoma State University-Stillwater. Exclusion criteria included the presence of chronic medical conditions other than obesity, pregnancy, postmenopausal status, or current or past use of lipid-lowering medications, tobacco, or illicit substances. Participants were categorized into one of four groups (n = 20 per group, evenly divided between females and males) based on BMI and relative body fat. The groups included NWL, NWO and OB. NWO was defined by a normal-weight BMI (18.5–24.9 kg/m^2^) and high body fat percentage (i.e., >25% M, >35% F), as previously described [17,22,23] and according to World Health Organization body fat percentage cutoffs [24]. NWL exhibited a normal-weight BMI (18.5–24.9 kg/m^2^) and low body fat (<25% M, <35% F). The OB group was defined by a BMI ≥ 30.0 kg/m^2^ and met the same body fat percentage criteria applied to NWO (>25% in males and >35% in females).

An a priori sample size estimation was not determined for this study. However, according to a post hoc power analysis (G*Power) based on our primary outcome (VO_2peak_) and our primary statistical approach (analysis of variance (ANOVA) with fixed effects), and including our observed results for each group, we have 99% power to detect group-based differences in aerobic capacity.

This study was part of two studies registered at ClinicalTrials.gov (NCT05008952, NCT05889767). All procedures were conducted in accordance with the Declaration of Helsinki and approved by the Oklahoma State University Institutional Review Board (IRB-20-339-STW).

### 2.2. Metabolic Outcome Assessment

A fasting blood sample was collected via an antecubital vein using a 21-gauge needle. The sample was then analyzed for metabolic syndrome risk factors (i.e., glucose, triglycerides, HDL) and other metabolic markers using the Piccolo Xpress clinical chemistry analyzer (Lipid Panel Plus discs; Abbott; Abbott Park, IL, USA). After blood collection, participants rested in a quiet, dark environment in the supine position for approximately 10 min before blood pressure was measured. Blood pressure was recorded twice using an automated cuff (Omron; Kyoto, Japan), with the average taken, by trained research staff. If the two readings differed by more than 10 mmHg for either systolic or diastolic pressure, a third measurement was performed, and the average of all readings was used.

### 2.3. Body Composition Assessment

Body composition (i.e., relative body fat, absolute fat mass, relative lean mass, absolute lean mass, and visceral adipose tissue (VAT) mass) was assessed using dual-energy X-ray absorptiometry (DXA; Hologic Horizon A; Hologic, Inc.; Marlborough, MA, USA). Relative VAT was calculated by dividing VAT mass by participant height. Waist circumference was measured by trained personnel using a Gulick tape measure. Participants abstained from exercise (24 h), alcohol (24 h), and caffeine (10 h) before the measurement.

### 2.4. Aerobic Capacity Assessment

Participants refrained from exercise (24 h), alcohol (24 h), and caffeine (10 h) prior to aerobic capacity testing. Peak oxygen uptake (VO_2peak_) was measured using a TrueOne 2400 metabolic cart (Parvo Medics; Sandy, UT, USA) during an incremental cycling test (Monark 928 E ergometer; Monark Sports and Medical; Vansbro, Sweden) to voluntary exhaustion. To account for variability in baseline fitness, two exercise protocols were implemented to achieve a consistent test duration across participants: cycling workload either increased 5 watts every 15 s or 5 watts every 20 s, after an initial workload of 25 watts. Protocols were based on responses from the international physical activity questionnaire short form [25] and informal discussions, as needed. Participants were instructed to maintain a pedaling cadence between 60 and 80 revolutions per minute throughout the test. Heart rate was continuously monitored (Polar H10; Polar Electro; Kempele, Finland), and rate of perceived exertion (RPE) was self-reported every minute. Testing ended when participants could no longer sustain a 60 revolutions per minute cadence for five consecutive revolutions or they reached self-determined volitional fatigue. VO_2_ was averaged every 15 s, and the highest 15 s average was used as the participant’s VO_2peak_. Given that VO_2peak_, as opposed to VO_2max_, was our primary endpoint, we did not attempt to verify our peak values as true VO_2max_ values through observation of a plateau in VO_2_ or certain thresholds of secondary criteria, such as respiratory exchange ratio, RPE, or heart rate. A plateau in VO_2_ with increasing workload is inconsistently observed [26,27], and data suggest that secondary criteria are not effective proxies for determining VO_2max_ [28]. An approach that has been recommended is to have participants perform a secondary validation test at a higher workload to confirm no greater VO_2_ values [29]. While rigorous, this validation test was not utilized in the present study due to the additional burden on participants, namely participants with obesity, a population that has reported discomfort with or fear of exercise [30,31].

### 2.5. Statistical Analyses

Data were checked for normality with the Shapiro–Wilk test. If data were not normally distributed, they were log-transformed prior to analysis. Student’s *t* test was used to test for differences in aerobic capacity between sexes, and one-way analysis of variance (ANOVA) was used to assess differences in aerobic capacity between body composition groups, irrespective of sex (i.e., NWL vs. NWO vs. OB). Two-way ANOVA with Sidak multiple comparison tests was used to test for differences between body composition groups and sexes with respect to participant characteristics, metabolic outcomes, body composition parameters, and exercise test outcomes. Pearson correlations were used to test for associations between body composition parameters and aerobic capacity in the full sample and stratified by sex. An alpha level of 0.05 was utilized as the threshold for statistical significance. All analyses were conducted in GraphPad Prism 10.2.2.

## 3. Results

Data for all variables were normally distributed, except for systolic blood pressure, total cholesterol, homeostatic model assessment of insulin resistance (HOMA-IR), and AST. These variables were log-transformed, after which they passed the Shapiro–Wilk test.

Participant characteristics are displayed in Table 1. Effect sizes for all ANOVAs are displayed in Appendix A. The OB group was significantly older than the NWL group (+7.05 years; *p* = 0.012). As expected, BMI was greater in OB compared to NWL (*p* < 0.0001) and NWO (*p* < 0.0001), but BMI was also greater in NWO than NWL (+2.18 kg/m^2^; *p* = 0.037). Waist circumference (+3.68 in; *p* = 0.008) was greater in NWO than NWL, with OB greater than both groups (*p* < 0.0001). Systolic blood pressure, diastolic blood pressure, triglycerides, and VLDL were all greater in OB compared to NWL and NWO, with no difference between NWL and NWO. Total cholesterol and LDL were all greater, and HDL lower, in OB compared to NWL, with NWO not being different from either group. The total-cholesterol-to-HDL ratio was greater in OB than NWO, which was greater than NWL. HOMA-IR was lower in NWL than NWO and OB, with no difference between NWO and OB. No body-composition-based group differences were observed in insulin, ALT, or AST.

There were few sex differences in participant characteristics within body composition phenotypes. NWL males exhibited greater systolic blood pressure than NWL females. In OB, males exhibited greater body mass and lower HDL. No other differences were observed.

Body composition outcomes are displayed in Table 2. OB exhibited greater absolute body fat compared to NWL and NWO, with no difference between NWL and NWO. Conversely, relative body fat was greater in NWO than NWL (+5.14%; *p* = 0.003), with OB being greater than both groups. Absolute lean mass was greater in OB than NWL and NWO. Relative lean mass was lower in NWO than NWL (−4.88%; *p* = 0.009), with OB being lower than both groups. VAT and relative VAT (VAT/height) were greater in OB than NWL, but NWO was not different from either group.

Relative body fat was greater in NWL females than NWL males (+8.98%; *p* = 0.0003) and greater in OB females than OB males (+9.02%; *p* = 0.0003), but there was no difference in body fat between NWO females and males (+0.72%; *p* = 0.982). Similarly, absolute and relative lean mass was greater in males than females in the NWL (+11.91 kg; +8.49%; *p*’s < 0.004) and OB groups (+18.77 kg; +8.83%; *p*’s < 0.0008), but not in NWO (*p*’s > 0.98).

Our exercise data are consistent with the notion that participants exercised to volitional fatigue. The mean RPE at peak exercise was 9.5 or greater (on a 10-point scale) for all groups and sub-groups. With only two exceptions, participants reached an RPE of 10 at peak exercise. Ninety percent of participants exhibited a respiratory exchange ratio of 1.1 or greater at peak exercise.

Across body composition phenotypes, females exhibited a lower VO_2peak_ (28.8 ± 8.8 mL/kg/min) than males (34.5 ± 11.8 mL/kg/min; *p* = 0.043) when normalized to body mass. This difference was also observed when normalized to lean mass (−6.5 mL/kg LM/min; *p* = 0.047) and fat mass (−63.5 mL/kg FM/min; *p* = 0.001).

Peak exercise parameters are displayed in Table 3. Peak expired ventilatory volume was greater in NWL than NWO (+26.51 L/min; *p* = 0.005), with OB being different from neither group. A group main effect was observed for peak tidal volume, but there were no significant post hoc pairwise comparisons. Peak power was greater in NWL compared to NWO (+66.44 W; *p* < 0.0001) and OB (+49.83 W; *p* = 0.0003), but NWO and OB were not different (*p* = 0.355). Peak respiratory exchange ratio, peak heart rate and peak rating of perceived exertion (RPE) were not different between groups, suggesting all groups exercised to a similar relative peak exercise intensity.

Sex differences were observed in peak ventilation and tidal volume in NWL, now, and OB. NWL males exhibited greater peak power than females (+65.76 W; *p* = 0.001), which was also observed in OB (+46.00 W; *p* = 0.024). Interestingly, there was no sex difference in peak power in NWO (+30.78 W; *p* = 0.218). No sex differences were observed in peak respiratory exchange ratio, heart rate, or RPE.

**Figure 1:**
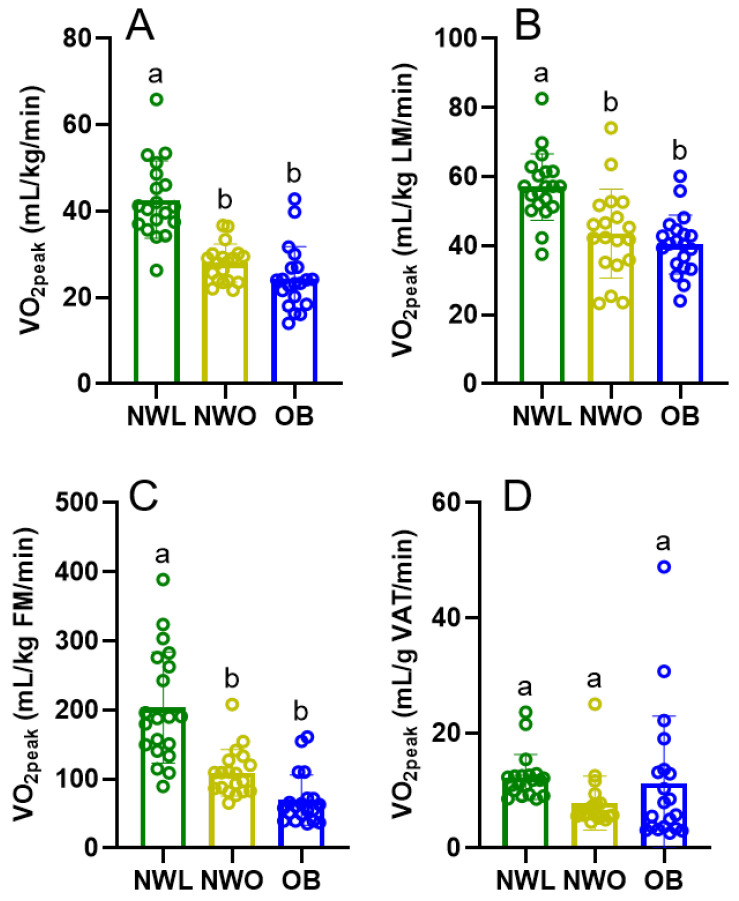
**VO_2peak_ outcomes by body composition phenotype.** Comparison of VO_2peak_ by body composition phenotype when VO_2peak_ is normalized to body mass (Panel (**A**)), lean mass (Panel (**B**)), fat mass (Panel (**C**)), and VAT mass (Panel (**D**)). Within each panel, bars with shared superscripts are not significantly different (*p* > 0.05). LM, lean mass; FM, fat mass; VAT, visceral adipose tissue.

Peak aerobic capacity metrics are displayed in Figure 1. NWL exhibited greater aerobic capacity than NWO and OB when VO_2peak_ was normalized by total mass, lean mass, and fat mass. Notably, there were no differences in aerobic capacity between NWO and OB across all VO_2peak_ analyses. There was also no difference in aerobic capacity between groups when normalized by VAT mass.

**Figure 2:**
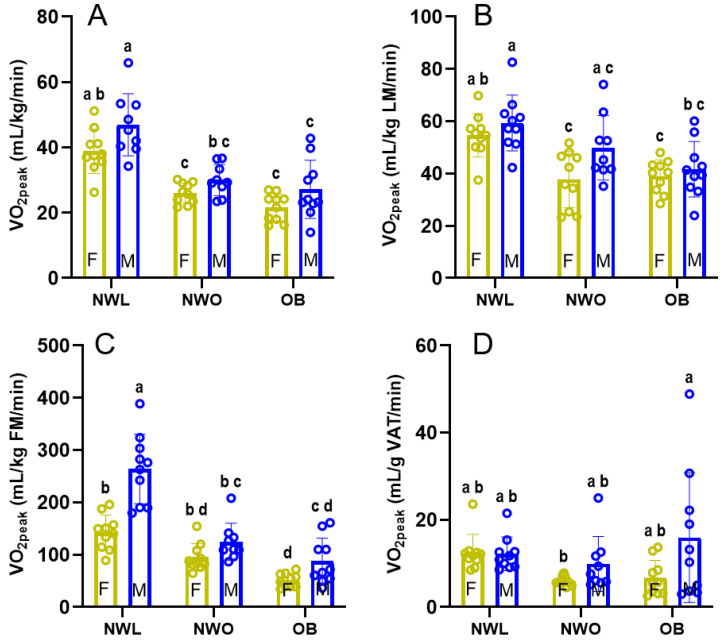
**VO_2peak_ outcomes by sex and body composition phenotype.** Comparison of VO_2peak_ by sex and body composition phenotype when VO_2peak_ is normalized to body mass (Panel (**A**)), lean mass (Panel (**B**)), fat mass (Panel (**C**)), and VAT mass (Panel (**D**)). Within each panel, bars with shared superscripts are not significantly different (*p* > 0.05). LM, lean mass; FM, fat mass; VAT, visceral adipose tissue.

With respect to sex-specific comparisons of aerobic capacity between body composition groups (Figure 2), NWL males and females exhibited greater VO_2peak_ compared to NWO and OB. Specifically, NWL males exhibited greater VO_2peak_ than NWO (+16.89 mL/kg/min; *p* < 0.0001) and OB (+19.75 mL/kg/min; *p* < 0.0001) males, and NWL females exhibited greater VO_2peak_ than NWO (+12.76 mL/kg/min; *p* = 0.001) and OB (+17.14 mL/kg/min; *p* < 0.0001) females. There were no differences in aerobic capacity normalized to body mass between NWO and OB. There were also no differences between sexes within each body composition phenotype.

When aerobic capacity was normalized to absolute lean mass (Figure 2B), NWL females presented with greater VO_2peak_ compared to NWO (+16.93 mL/kg LM/min; *p* = 0.005) and OB (+15.67 mL/kg LM/min; *p* = 0.011) females. However, there was no difference between NWL and NWO males (*p* = 0.322), suggesting that differences in lean mass may not drive aerobic capacity differences between NWL and NWO males. There were no sex differences within body composition phenotypes.

Aerobic capacity is also displayed relative to absolute fat mass in Figure 2C. VO_2peak_ was not different between NWL and NWO females, although NWL was greater than OB (+91.33 mL/kg FM/min; *p* < 0.0001). However, in males, NWL exhibited greater VO_2peak_ than NWO (+139.4 mL/kg FM/min; *p* < 0.0001) and OB (+176.4 mL/kg FM/min; *p* < 0.0001) when normalizing by absolute fat mass, indicating fat mass may explain more variation in VO_2peak_ by body composition phenotype in males than females. NWL females exhibited significantly lower VO_2peak_ than NWL males (−121.2 mL/kg FM/min; *p* < 0.0001) when normalized to body fat, a trend that was not observed in NWO or OB.

To explore the relationship between VAT specifically and aerobic capacity, VO_2peak_ is normalized to VAT mass in Figure 2D. However, the only observed difference was that OB males were higher than NWO females.

**Figure 3:**
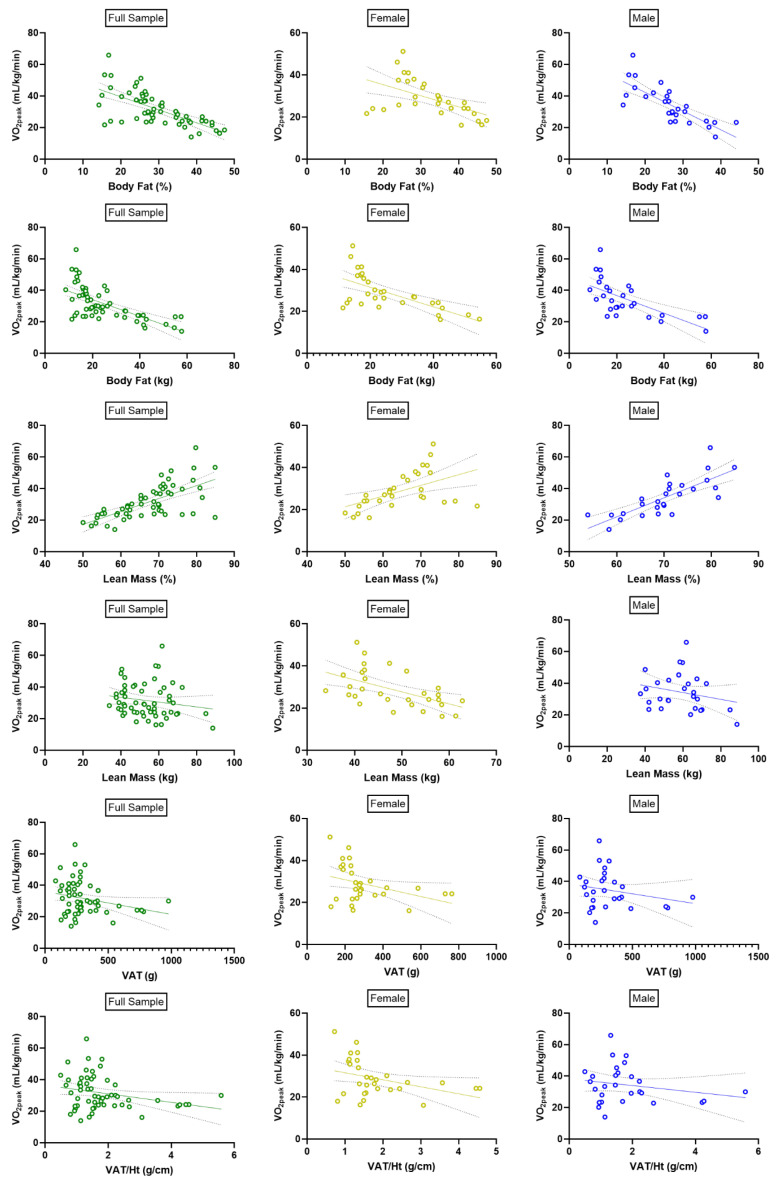
**Correlations between body composition parameters and aerobic capacity.** Associations of VO_2peak_ with relative body fat (first row), absolute body fat (second row), relative lean mass (third row), absolute lean mass (fourth row), VAT (fifth row), and relative VAT (sixth row) in the full sample (left column), females only (middle column), and males only (right column). VAT, visceral adipose tissue.

Correlations between body composition parameters and aerobic capacity are displayed in Figure 3. In the full sample, significant inverse correlations were observed between VO_2peak_ normalized to body mass and relative body fat (*r* = −0.674; *p* < 0.0001), absolute body fat (*r* = −0.649; *p* < 0.0001), and relative VAT (*r* = −0.270; *p* = 0.041), whereas a significant positive correlation was observed with relative lean mass (*r* = 0.662; *p* < 0.0001). There was no significant correlation between aerobic capacity and absolute lean mass (*r* = −0.171; *p* = 0.199) or absolute VAT mass (*r* = −0.254; *p* = 0.055).

In females only, there was a significant inverse correlation between VO_2peak_ and relative fat mass (*r* = −0.526; *p* = 0.003), absolute fat mass (*r* = −0.653; *p* < 0.0001), absolute lean mass (*r* = −0.534; *p* = 0.002), absolute VAT (*r* = −0.364; *p* = 0.048), and relative VAT (*r* = −0.368; *p* = 0.046). There was a significant positive correlation between VO_2peak_ and relative lean mass (*r* = 0.503; *p* = 0.005).

In males only, there was a significant negative correlation between aerobic capacity and relative fat mass (*r* = −0.777; *p* < 0.0001) and absolute fat mass (*r* = −0.664; *p* = 0.0001), whereas a significant positive correlation was observed between VO_2peak_ and relative lean mass (*r* = 0.776; *p* < 0.0001). There were no significant correlations between aerobic capacity and absolute lean mass (*r* = −0.240; *p* = 0.220), absolute VAT (*r* = −0.225; *p* = 0.250), or relative VAT (*r* = −0.214; *p* = 0.275) in males.

## 4. Discussion

In the present work, we aimed to evaluate sex differences in aerobic capacity across three body composition phenotypes: NWL, NWO, and OB. We hypothesized that NWL would exhibit greater aerobic capacity than NWO and OB, and that this would be observed in both female- and male-specific analyses. We also predicted that females would exhibit a lower aerobic capacity than males within each body composition phenotype. Our hypotheses were largely supported, as we observed greater VO_2peak_ in NWL than NWO and OB, and that NWO and OB were not different. This was generally observed in both males and females, although the difference between NWL and NWO was negated in males when normalizing VO_2peak_ by lean mass and negated in females when normalizing by fat mass. Contrary to our hypotheses, we generally did not observe a difference in VO_2peak_ between males and females of the same body composition phenotype.

Our work is generally consistent with the limited literature on aerobic capacity in NWO. Zhang and colleagues studied physical fitness in 383 young adults, 77 of whom had NWO, via a 10 min endurance running test, counter-movement jumps, and a shuttle run test [15]. NWO participants displayed lower measures of fitness on all three fitness tests than their NWL counterparts. The authors observed that low skeletal muscle mass accounted for the lower fitness outcomes in both males and females. In a secondary data analysis, Bellissimo et al. also studied aerobic capacity in NWO, observing lower VO_2max_ in people with NWO than NWL, but greater aerobic capacity in people with NWO than a group with overweight and obesity [18]. In sex-specific comparisons, NWO females exhibited lower VO_2max_ than lean females but greater than females with overweight and obesity, whereas males with NWO exhibited VO_2max_ similar to males with overweight and obesity and less than lean males. In a recent study from our laboratory, we observed significantly lower VO_2peak_ in NWO compared to a NWL control group, and similar VO_2peak_ relative to metabolically healthy obesity and metabolically unhealthy obesity groups [17]. This trend was evident when VO_2peak_ was normalized to total body mass, lean mass, and fat mass. Our findings in the present study agree with those of past studies, as we observed that VO_2peak_ in NWO was lower than that in NWL and similar to OB when aerobic capacity was normalized to body mass—a finding that was evident in both female- and male-stratified comparisons. Additionally, we observed this trend when aerobic capacity was normalized to fat mass and lean mass. It appears that the previously reported and presently observed lower aerobic capacity of people with NWO is not fully explained by either elevated body fat or limited lean mass, but likely contributes to the elevated cardiovascular disease risk observed in this population [13]. In support of this, studies have consistently demonstrated that cardiorespiratory fitness is similar between NWO and overt obesity.

Men are generally considered to present with a greater aerobic capacity than women. A study of 3816 healthy men and women observed an approximately +8 mL/kg/min difference between men and women [20]. Recent work suggests this may be largely explained by differences in blood volume and oxygen carrying capacity between sexes [32]. However, body composition no doubt has a significant influence on aerobic capacity, given the functional differences between fat and muscle in oxygen consumption and work production during exercise. Indeed, a recent study from the same laboratory observed that total and leg-specific lean mass were strong determinants of aerobic capacity, independent of relative fat mass [33]. Given documented differences in body composition between men and women [19], it is likely that differences in lean mass and fat mass between sexes account for some variation in aerobic capacity. This agrees with our findings, as we observed lower aerobic capacity in females compared to males when VO_2peak_ was normalized by total mass, lean mass, and fat mass.

Little is known about sex differences in aerobic capacity in novel body composition phenotypes like NWO. The study conducted by Zhang et al. referenced earlier reported fitness results stratified by sex, but did not make statistical comparisons between sexes or explore the influence of body composition differences [15]. Similarly, Bellissimo did not compare VO_2max_ between men and women with NWO, although descriptively, aerobic capacity was lower in females than males [18]. Thus, to our knowledge, the present study is the first to investigate sex differences in aerobic capacity in people with NWO, and to evaluate the influence of body composition in this relationship. Contrary to our expectation, we did not observe differences in aerobic capacity between females and males, regardless of whether VO_2peak_ was normalized by total mass, lean mass, fat mass, or VAT mass. This finding may be explained in part by similar body composition indices between NWO females and males. Specifically, there was no difference in absolute body fat, relative body fat, absolute lean mass, relative lean mass, absolute VAT, or relative VAT (all *p*’s ≥ 0.980). On the other hand, we observed significant sex differences in relative fat mass, absolute lean mass, and relative lean mass in the other groups. It is interesting to observe that, despite well-documented sex differences in body composition [34], these differences seem to be absent in the NWO phenotype. Given that women have more essential fat than men [35], this would seem to bode poorly for NWO men. However, this finding and its potential implications merit additional exploration.

We also studied correlations between aerobic capacity and body composition in the full sample and stratified by men and women. In the full sample, we observed significant inverse associations between aerobic capacity and relative body fat, absolute body fat, and relative VAT, and a positive association with relative lean mass. However, the relationship between body composition and aerobic capacity appears to be more consistent in females than males. Females exhibited a significant inverse correlation between VO_2peak_ and body fat (absolute and relative), absolute lean mass, and VAT mass (absolute and relative), as well as a positive correlation with relative lean mass. On the other hand, males did not exhibit a significant association between VO_2peak_ and absolute lean mass, absolute VAT, or relative VAT. The physiologic cause of this apparent sex difference in the relationship between body composition and aerobic capacity is unclear and warrants future investigation.

A few considerations should be made when interpreting our data. First, our assessment of aerobic capacity would have been strengthened by the inclusion of a validation test to verify that VO_2max_ was reached, as has been previously recommended [29]. Second, our work would be enhanced by mechanistic insights into how muscle or fat tissue specifically impacts determinants of aerobic capacity (e.g., cardiac output, mitochondrial density) in females and males with NWO. Third, despite successfully recruiting the target sample size into each group (NWL, NWO, and OB), we nevertheless observed a significant difference in BMI between NWL and NWO. Thus, although all participants in both groups had a normal-weight BMI, NWO participants had a slightly higher BMI than NWL participants, which should be taken into consideration. Fourth, we did not conduct an a priori sample size calculation. However, a post hoc power analysis suggests we were sufficiently powered to detect group differences in aerobic capacity based on ANOVA. Finally, due to limited statistical power, our analyses do not include statistical adjustments for age or ethnicity, which are factors that can influence body composition [36,37] and aerobic capacity [38,39]. It is also worth noting that our OB group was significantly older than our NWL group.

## 5. Practical Applications

This study highlights the importance of recognizing and addressing NWO as a distinct body composition phenotype associated with low aerobic capacity, comparable to that observed in traditional obesity. Healthcare professionals should consider screening for elevated body fat even in individuals with normal BMI, as these individuals may have hidden cardiometabolic risks. Interventions targeting aerobic fitness, such as structured exercise programs, may be particularly effective in reducing these risks in both men and women with NWO or OB. Given the strong inverse correlation between aerobic capacity and relative body fat, which was especially consistent in females, strategies targeting the improvement of cardiorespiratory fitness may yield significant improvements in cardiometabolic health. Additionally, the sex-specific differences observed in the relationship between visceral adiposity and aerobic capacity suggest that individualized approaches may be needed for males. These findings underscore the need for a broader understanding of body composition beyond BMI in clinical and public health settings to better tailor preventive and therapeutic strategies for reducing cardiometabolic risk.

## 6. Conclusions

We observed significantly lower aerobic capacity in NWO compared to NWL counterparts, and similar aerobic capacity to OB counterparts. This was observed in both females and males. Contrary to our hypothesis, we did not observe sex differences in body composition within specific body composition phenotypes. However, we did observe significant correlations between body composition and aerobic capacity across groups, a finding that was more consistent in females and males. Taken together, our work adds new data to the bodies of evidence regarding the influence of body composition on aerobic capacity, including the NWO phenotype, and fills a gap regarding potential sex differences in aerobic capacity in NWO.

## Figures and Tables

**Table 1:** **Participant characteristics.** Sex P columns indicate *p* values comparing females and males within a given body composition group. Group P indicates group effect *p* values for the total sample (i.e., inclusive of both sexes). In Total sample columns, cells within a row with shared superscripts are not significantly different (*p* > 0.05). N, sample size; BMI, body mass index; SBP, systolic blood pressure; DBP, diastolic blood pressure; ^, data were log-transformed prior to analysis; values in the table reflect raw measurements (to aid in interpretation), but *p* values are based on analysis of transformed data.

	NWL				NWO				OB				Group P
	Total	Female	Male	Sex P	Total	Female	Male	Sex P	Total	Female	Male	Sex P	
**N**	20	10	10	-	20	10	10	-	20	10	10	-	-
**Age (years)**	30.4 ± 7.0 ^a^	29.5 ± 7.0	31.3 ± 7.3	0.933	33.8 ± 7.1 ^ab^	33.9 ± 8.9	33.6 ± 5.3	0.999	37.5 ± 8.4 ^b^	40.3 ± 8.0	34.6 ± 8.1	0.259	**0.017**
**Mass (kg)**	67.3 ± 9.2 ^a^	62.8 ± 8.0	71.8 ± 8.4	0.226	70.9 ± 8.7 ^a^	64.9 ± 7.1	76.9 ± 5.3	0.064	104.4 ± 18.5 ^b^	96.0 ± 12.0	112.8 ± 20.5	**0.005**	**0.0001**
**BMI (kg/m^2^)**	22.0 ± 1.6 ^a^	21.6 ± 1.6	22.3 ± 1.6	0.896	24.2 ± 1.2 ^b^	24.6 ± 0.9	23.7 ± 1.3	0.861	35.6 ± 4.2 ^c^	35.6 ± 4.0	35.5 ± 4.6	0.999	**0.0001**
**Waist Circ. (in)**	28.7 ± 2.1 ^a^	27.9 ± 2.2	29.6 ± 1.8	0.691	32.4 ± 2.3 ^b^	31.1 ± 2.2	33.7 ± 1.6	0.324	41.8 ± 5.9 ^c^	40.2 ± 6.4	43.5 ± 5.1	0.137	**0.0001**
**SBP (mmHg) ^**	109.9 ± 11.8 ^a^	103.5 ± 10.3	116.4 ± 9.7	**0.031**	111.6 ± 9.5 ^a^	106.5 ± 7.5	116.7 ± 8.8	0.138	125.8 ± 15.6 ^b^	125.3 ± 15.8	126.3 ± 16.2	0.997	**0.0002**
**DBP (mmHg)**	73.1 ± 8.0 ^a^	73.0 ± 5.4	73.2 ± 10.2	0.999	76.0 ± 8.4 ^a^	77.0 ± 4.6	75.0 ± 11.2	0.955	86.4 ± 12.2 ^b^	90.3 ± 12.9	82.4 ± 10.6	0.202	**0.0002**
**Chol. (mg/dL) ^**	154.8 ± 35.5 ^a^	150.9 ± 25.0	158.6 ± 44.8	0.988	174.2 ± 35.2 ^ab^	175.9 ± 39.2	172.4 ± 32.8	0.996	183.3 ± 32.5 ^b^	193.9 ± 37.8	172.6 ± 23.4	0.554	**0.021**
**HDL (mg/dL)**	64.5 ± 13.7 ^a^	66.2 ± 11.8	62.8 ± 15.8	0.876	58.3 ± 9.2 ^ab^	61.9 ± 9.4	54.7 ± 7.9	0.401	50.2 ± 12.0 ^b^	56.5 ± 10.3	43.9 ± 10.5	**0.044**	**0.0008**
**TG (mg/dL)**	64.5 ± 17.5 ^a^	72.7 ± 16.9	56.3 ± 14.6	0.861	95.8 ± 39.6 ^a^	102.0 ± 47.8	89.6 ± 30.8	0.933	144.8 ± 76.5 ^b^	147.5 ± 86.7	142.0 ± 69.5	0.994	**0.0001**
**LDL (mg/dL)**	77.2 ± 29.6 ^a^	70.2 ± 21.0	84.2 ± 36.1	0.601	96.7 ± 29.4 ^ab^	93.4 ± 34.3	99.9 ± 24.9	0.937	104.3 ± 22.8 ^b^	108.1 ± 25.5	100.4 ± 20.5	0.901	**0.01**
**TC/HDL**	2.45 ± 0.48 ^a^	2.34 ± 0.44	2.55 ± 0.53	0.882	3.02 ± 0.58 ^b^	2.90 ± 0.76	3.13 ± 0.32	0.851	3.77 ± 0.98 ^c^	3.50 ± 0.76	4.04 ± 1.13	0.254	**0.0001**
**VLDL (mg/dL)**	12.9 ± 3.5 ^a^	14.5 ± 3.3	11.2 ± 2.9	0.855	19.2 ± 8.0 ^a^	20.6 ± 9.5	17.8 ± 6.3	0.905	28.8 ± 15.1 ^b^	29.4 ± 17.3	28.2 ± 13.5	0.991	**0.0001**
**Gluc. (mg/dL)**	91.0 ± 8.4 ^a^	93.5 ± 7.2	88.5 ± 9.1	0.413	99.7 ± 6.5 ^b^	97.7 ± 6.7	101.7 ± 5.9	0.599	103.6 ± 9.2 ^b^	106.6 ± 11.0	100.6 ± 6.2	0.259	**0.0001**
**Insulin (mg/dL)**	1.21 ± 0.81	1.11 ± 0.44	1.34 ± 1.10	0.999	3.40 ± 5.28	4.80 ± 7.29	2.00 ± 1.16	0.226	3.93 ± 2.99	4.23 ± 3.13	3.60 ± 2.99	0.974	0.055
**HOMA-IR ^**	0.27 ± 0.18 ^a^	0.25 ± 0.09	0.29 ± 0.25	0.999	0.83 ± 1.25 ^b^	1.15 ± 1.72	0.50 ± 0.30	0.298	1.03 ± 0.88 ^b^	1.17 ± 1.01	0.88 ± 0.73	0.862	**0.001**
**ALT (U/L)**	27.6 ± 13.9	21.6 ± 4.0	33.5 ± 17.6	0.285	32.0 ± 21.1	27.6 ± 18.9	36.3 ± 23.3	0.551	34.8 ± 14.3	27.9 ± 7.3	41.1 ± 16.4	0.224	0.404
**AST (U/L) ^**	32.9 ± 12.4	29.4 ± 6.0	36.3 ± 16.2	0.483	31.5 ± 14.3	32.1 ± 18.7	30.9 ± 8.8	0.994	29.8 ± 6.9	28.6 ± 5.4	30.9 ± 8.2	0.963	0.802

Bolded *p* values are significant (*p* < 0.05).

**Table 2:** **Body composition outcomes.** Sex P columns indicate *p* values comparing females and males within a given body composition group. Group P indicates group effect *p* values for the total sample (i.e., inclusive of both sexes). In Total sample columns, cells within a row with shared superscripts are not significantly different (*p* > 0.05). N, sample size; VAT, visceral adipose tissue; VAT/Ht; VAT relative to height.

	NWL				NWO				OB				Group P
	Total	Female	Male	Sex P	Total	Female	Male	Sex P	Total	Female	Male	Sex P	
**N**	20	10	10	-	20	10	10	-	20	10	10	-	-
**Body fat (kg)**	15.1 ± 3.4 ^a^	17.3 ± 2.9	12.9 ± 2.3	0.399	18.9 ± 3.8 ^a^	18.7 ± 4.8	19.0 ± 2.6	0.999	39.9 ± 10.7 ^b^	41.1 ± 7.5	38.7 ± 13.4	0.811	**0.0001**
**Body fat (%)**	22.5 ± 5.4 ^a^	27.0 ± 2.5	18.0 ± 3.2	**0.0003**	27.7 ± 5.7 ^b^	28.0 ± 8.0	27.3 ± 2.2	0.982	37.9 ± 6.7 ^c^	42.4 ± 3.3	33.4 ± 6.3	**0.0003**	**0.0001**
**Lean mass (kg)**	50.4 ± 9.1 ^a^	44.4 ± 6.0	56.3 ± 7.8	**0.004**	47.7 ± 9.2 ^a^	47.1 ± 10.7	48.4 ± 7.9	0.98	62.3 ± 11.7 ^b^	52.9 ± 5.1	71.7 ± 8.3	**0.0001**	**0.0001**
**Lean mass (%)**	74.3 ± 5.5 ^a^	70.1 ± 2.7	78.5 ± 4.0	**0.001**	69.4 ± 6.1 ^b^	69.2 ± 8.4	69.6 ± 2.7	0.997	59.6 ± 6.5 ^c^	55.2 ± 3.1	64.0 ± 6.1	**0.0007**	**0.0001**
**VAT (g)**	241 ± 53 ^a^	203 ± 39	280 ± 36	0.695	287 ± 80 ^ab^	285 ± 36	290 ± 110	0.999	405 ± 280 ^b^	420 ± 231	390 ± 334	0.976	**0.013**
**VAT/Ht (g/cm)**	1.38 ± 0.28 ^a^	1.19 ± 0.20	1.57 ± 0.21	0.779	1.69 ± 0.45 ^ab^	1.76 ± 0.24	1.61 ± 0.60	0.98	2.36 ± 1.60 ^b^	2.54 ± 1.38	2.18 ± 1.86	0.803	**0.009**

Bolded *p* values are significant (*p* < 0.05).

**Table 3:** **Peak exercise parameters.** Sex P columns indicate *p* values comparing females and males within a given body composition group. Group P indicates group effect *p* values for the total sample (i.e., inclusive of both sexes). In Total sample columns, cells within a row with shared superscripts are not significantly different (*p* > 0.05). N, sample size; V_E_, expire ventilation; V_T_, tidal volume; RER, respiratory exchange ratio; HR, heart rate; RPE, rating of perceived exertion.

	NWL				NWO				OB				Group P
	Total	Female	Male	Sex P	Total	Female	Male	Sex P	Total	Female	Male	Sex P	
**N**	20	10	10	-	20	10	10	-	20	10	10	-	-
**Peak V_E_ (L/min)**	102.9 ± 31.5 ^a^	83.8 ± 18.9	124.1 ± 29.4	**0.003**	76.7 ± 27.6 ^b^	62.7 ± 16.8	92.2 ± 29.6	**0.038**	91.1 ± 30.2 ^ab^	76.3 ± 18.2	106.0 ± 33.1	**0.03**	**0.008**
**Peak V_T_ (L)**	2.60 ± 0.56	2.25 ± 0.24	2.99 ± 0.56	**0.006**	2.23 ± 0.70	1.85 ± 0.55	2.64 ± 0.63	**0.003**	2.62 ± 0.66	2.16 ± 0.38	3.08 ± 0.55	**0.0004**	**0.04**
**Peak RER**	1.23 ± 0.14	1.19 ± 0.14	1.27 ± 0.12	0.383	1.20 ± 0.08	1.19 ± 0.07	1.22 ± 0.09	0.797	1.18 ± 0.11	1.15 ± 0.07	1.20 ± 0.14	0.702	0.463
**Peak HR (bpm)**	168 ± 22	173 ± 6	162 ± 32	0.715	165 ± 25	158 ± 27	172 ± 23	0.508	163 ± 23	156 ± 21	170 ± 24	0.443	0.842
**Peak Power (W)**	217.1 ± 61.3 ^a^	186.0 ± 20.0	251.7 ± 73.8	**0.001**	151.6 ± 28.4 ^b^	137.0 ± 19.5	167.8 ± 28.8	0.218	169.0 ± 36.8 ^b^	146.0 ± 23.9	192.0 ± 33.4	**0.024**	**0.0001**
**Peak RPE (au)**	9.9 ± 0.2	10.0 ± 0.0	9.9 ± 0.3	0.978	10.0 ± 0.0	10.0 ± 0.0	10.0 ± 0.0	0.999	9.8 ± 1.1	10.0 ± 0.0	9.5 ± 1.6	0.275	0.477

Bolded *p* values are significant (*p* < 0.05).

## Data Availability

The datasets presented in this article are not readily available because the Institutional Review Board at Oklahoma State University has not authorized the authors to share these data publicly. Requests to access the datasets should be directed to the corresponding author (S. Emerson).

## Appendix A

**Table A1:** **ANOVA effect sizes.** Effect sizes (η^2^) are displayed for each variable, including group-, sex- and interaction-based effects. Effect sizes were calculated as the sum of squared of the respective domain (i.e., group, sex or interaction) divided by the total sum of squares. HOMA-IR, homeostatic model assessment of insulin resistance; VAT, visceral adipose tissue; RER, respiratory exchange ration; RPE, rating of perceived exertion. LM, lean mass; FM, fat mass.

	Group Effect Size (η^2^)	Sex Effect Size (η^2^)	Interaction Effect Size (η^2^)
Age	0.13	0.01	0.04
Mass	0.64	0.09	0.01
BMI	0.84	0.00	0.00
Waist circumference	0.68	0.04	0.00
Systolic BP	0.25	0.08	0.03
Diastolic BP	0.26	0.02	0.02
Total cholesterol	0.11	0.01	0.03
HDL	0.21	0.09	0.02
Triglycerides	0.31	0.01	0.00
LDL	0.15	0.01	0.02
TC/HDL	0.38	0.03	0.01
VLDL	0.31	0.01	0.00
Glucose	0.31	0.02	0.06
Insulin	0.10	0.02	0.03
HOMA-IR	0.12	0.03	0.02
ALT	0.03	0.11	0.00
AST	0.01	0.01	0.02
Body fat (kg)	0.73	0.01	0.01
Body fat (%)	0.55	0.13	0.05
Lean mass (kg)	0.29	0.21	0.10
Lean mass (%)	0.52	0.12	0.05
VAT	0.15	0.00	0.02
VAT/Height	0.16	0.00	0.02
Peak ventilation	0.12	0.29	0.01
Peak tidal volume	0.07	0.40	0.00
Peak RER	0.03	0.06	0.00
Peak heart rate	0.01	0.02	0.06
Peak workload	0.30	0.21	0.02
Peak RPE	0.03	0.02	0.03
VO_2peak_ (mL/kg/min)	0.57	0.08	0.01
VO_2peak_ (mL/kg LM/min)	0.33	0.07	0.03
VO_2peak_ (mL/kg FM/min)	0.53	0.16	0.07
VO_2peak_ (mL/g VAT/min)	0.06	0.08	0.07
